# Antimicrobial Resistance Analysis of Clinical *Escherichia coli* Isolates in Neonatal Ward

**DOI:** 10.3389/fped.2021.670470

**Published:** 2021-05-25

**Authors:** Dan Wu, Yijun Ding, Kaihu Yao, Wei Gao, Yajuan Wang

**Affiliations:** ^1^Department of Neonatology, Beijing Children's Hospital, Capital Medical University, National Center for Children's Health, Beijing, China; ^2^Laboratory of Dermatology, Beijing Pediatric Research Institute, Beijing Children's Hospital, Capital Medical University, National Center for Children's Health, Beijing, China; ^3^Department of Neonatology, Children's Hospital, Capital Institute of Pediatrics, Beijing, China

**Keywords:** *Escherichia coli*, antimicrobial drug resistance, E-test, ESBL, neonate

## Abstract

**Background:** *Escherichia coli* (*E. coli*) column for one of the most common pathogens causing neonatal infections. The emergence of antibiotic-resistant bacteria is a major cause of treatment failure in infected newborns. The purpose of this study was to describe antibiotic and multidrug resistance of *E. coli* strains isolated from neonates with infection throughout the years 2009–2011.

**Methods:** The antimicrobial susceptibility testing of *E. coli* strains to selected antibiotics was assessed using the E-test technique on the Mueller-Hinton agar. The antimicrobial tests included ceftazidime, cefuroxime, cefatriaxone, amoxicillin, amoxicillin-clavulanic acid, cefoperazone- sulbactam, meropenem, gentamicin, ciprofloxacin, and sulfonamides.

**Results:** A total of 100 *E. coli* strains were isolated from sputum (*n* = 78), blood (*n* = 10), cerebrospinal fluid (*n* = 5), and umbilical discharge (*n* = 7) samples of hospitalized neonates at the Beijing Children's Hospital. The highest rate of *E. coli* resistance was found in amoxicillin (85%), followed by cefuroxime (65%), and cefatriaxone (60%), respectively. A total of 6 and 5% of all isolates were only resistant to amoxicillin/clavulanic acid and cefoperazone -sulbactam. The rates of resistance to ceftazidime, gentamicin, ciprofloxacin, and sulfonamides were 31, 20, 33, and 47%, respectively. All isolates were susceptible to meropenem. Approximately 26% of all *E. coli* isolates were multidrug-resistant. The detection rate of ESBL-Producing *E. coli* was 55%.

**Conclusions:** Multi-drug-resistant *E. coli* has become an important and complex problem in clinical treatment, and it is thus essential to monitor *E. coli* resistance in neonates.

## Introduction

Newborns suffer high rates of mortality due to infectious diseases (1). Neonatal sepsis is the third leading cause of neonatal mortality, after prematurity and intrapartum-related complications (or birth asphyxia) (2).

*Escherichia coli* (*E. coli*) is the most common Gram-negative bacterium responsible for a variety of diseases as a result of community and hospital acquired clinically significant blood stream infections (BSIs), and constitutes a major cause o-f mortality from these infections at all ages. Pathogenic *E. coli* strains can be divided according to infection site into intestinal and extraintestinal (ExPEC). In recent years, many scholars in North America and Europe have continuously reported ExPEC with serious pathogenicity. A contemporary collection comprising 12,737 strains from pediatric patients (<18 years) isolated over a 7-year period (1998–2004) from 52 sentinel hospitals in North America showed that *E. coli* ranks in the top 6 pediatric pathogens (3) and that ExPEC is the leading cause of infections in neonates among gram-negative bacteria (4, 5).

In the past few years, antibiotics helped saving a significant number of lives and reduced the illness of several million people across the world (3). However, the remarkable benefits of antimicrobials in reducing morbidity and mortality rates have been challenged by the emergence of drug resistant strains in recent years, a more prevalent problem in developing countries for a variety of reasons (2, 3). The emergence and rapid spread of extended-spectrum cephalosporin and carbapenem resistance in *Enterobacteriaceae* is becoming a global health challenge. In addition, antibiotic-resistant *E. coli* are also increasing and becoming a major threat for global human health.

The emergence of multidrug-resistant *E. coli*, has been observed in various countries over the past decades. The increasing resistance to cephalosporins, especially the parallel rise in the frequency of multidrug-resistant *E. coli*, constitutes an increasing concern for the treatment of *E. coli* disease. The predominant mechanism of resistance to β-lactam antibiotics in *E. coli* is the production of plasmid-borne extended-spectrum β-lactamases (ESBLs). Since the first report in the early 1980s, ESBL-producing organisms have become widespread throughout the world (6). The ESBL genes are frequently encoded on transferable plasmids that encode resistance genes, and the acquisition of these resistant genes by commensal or fecal isolates leads, in turn, to multidrug resistant (MDR) pathogens.

To the best of our knowledge, there are limited data regarding *E. coli* antibiotic susceptibility in neonatal invasive diseases worldwide, particularly in China. In this study, we aim to investigate antibiotic susceptibility and multi-drug resistance of *E. coli* isolates that cause neonatal infections in order to provide a basis for clinical treatment of *E. coli* infections.

## Materials and Methods

### Study Design

**Figure 1:**
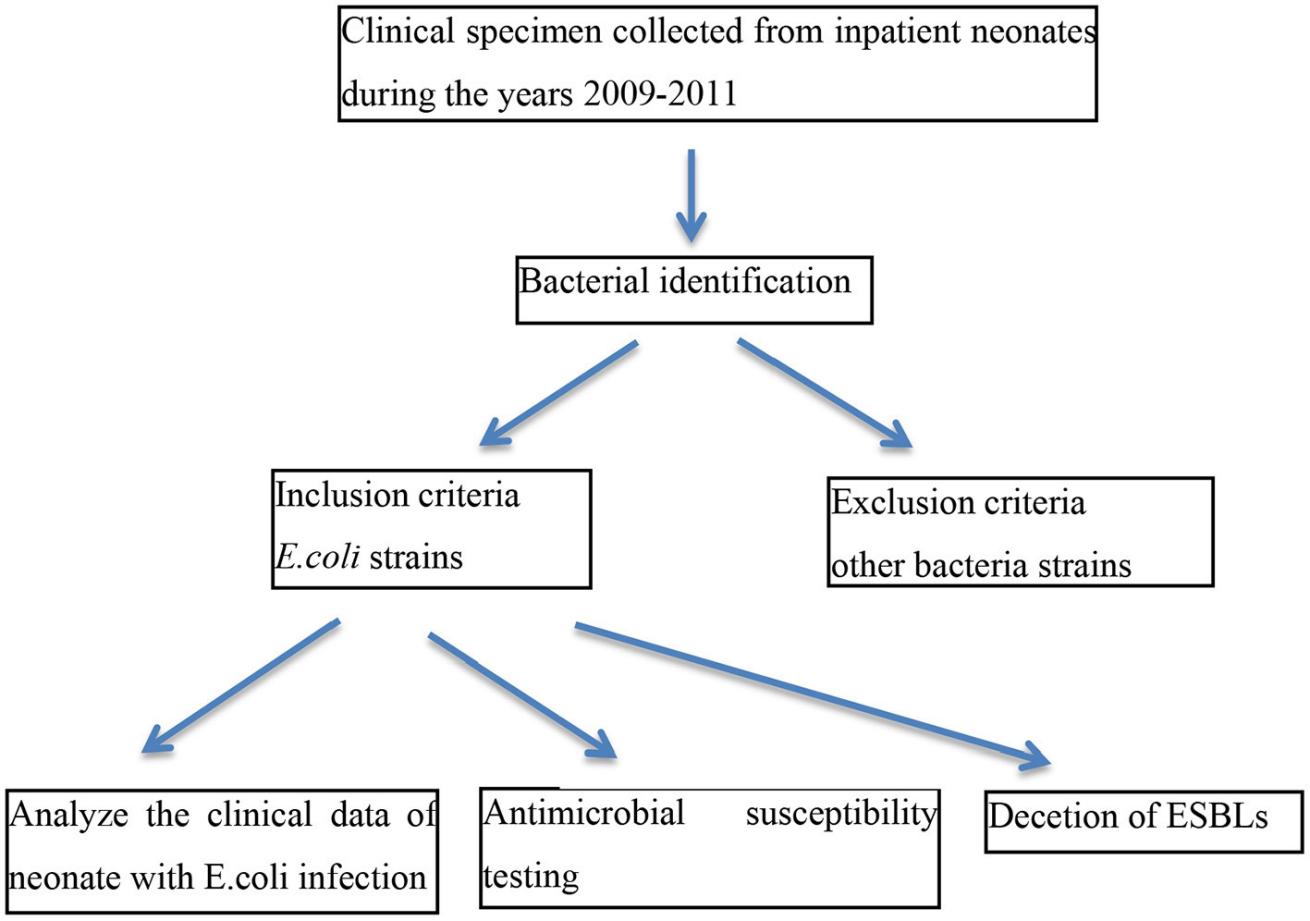
Experimental procedure. The experimental procedure included inclusion and exclusion criteria was shown.

This study was performed at the Beijing Children's Hospital, a tertiary facility with 110 beds in the neonatal unit, which handles more than 3,000 inpatient neonates per year. sputum, blood and/or cerebrospinal fluid (CSF) samples were taken from inpatient neonates diagnosed with pneumonia, sepsis and/or meningitis. Patients aged <28 days with *E. coli* positive cultures were enrolled, other bacteria strains were excluded. The study period spanned the years 2009–2011. This study was approved by the ethics committee of Beijing Children's Hospital, in accordance with the Declaration of Helsinki. The experimental procedure is shown in Figure 1.

### Bacterial Identification

*E. coli* species identification was performed using an ATB automatic bacterial identification instrument (France merrier company), a VITEK automatic biological analysis system (Biomerier China company) or a French merieres Merieux API system.

### Antimicrobial Susceptibility Testing of *E. coli* and Detection of ESBLs

The antimicrobial susceptibility testing of *E. coli* strains to selected antibiotics was assessed with the E-test technique (AB Biodisk-solana, Sweden) on the Mueller-Hinton agar (Becton Dickinson). The antimicrobial tests included were ceftazidime, cefuroxime, cefatriaxone, amoxicillin, amoxicillin-clavulanic acid, cefoperazone—sulbactam, meropenem, gentamicin, ciprofloxacin, and sulfonamides. The minimal inhibitory concentration (MIC) values of the antimicrobial agents selected for this study were determined by an agar dilution technique on Mueller-Hinton agar (Oxoid) according to the Clinical and Laboratory Standards Institute (CLSI) recommendations (7). The *E. coli* strain ATCC 25922 was used for routine quality-control assays. Multi-drug resistant (MDR) *E. coli* was defined as a strain showing non-susceptibility to at least one agent in three or more antimicrobial categories.

### Detection of ESBLs

The MICs of oxyimino-β-lactams and clavulanic acid were determined at a fixed concentration of 4 mg/l. The production of *E. coli* Extended-spectrum beta-lactamases (ESBLs) was determined using the double-disk synergy test (DDST). Specifically, this was performed with cefotaxime (30 μg) and ceftazidime (30 μg) disks placed at a distance of 20 mm (center to center) from the amoxicillin-clavulanic acid disk (20/10 μg). Moreover, cefpodoxime (10 μg), and aztreonam (30 μg) disks were added to increase the sensitivity of the DDST. A cefepime (30 μg) disk was placed in the same culture medium in order to improve the detection of ESBL during the simultaneous stable hyperproduction of an AmpC beta-lactamase. The test result was considered positive when an enhancement of the inhibition zone around at least one of the antibiotic disks (cefotaxime, ceftazidime, cefpodoxime, aztreonam, or cefepime) toward the clavulanic acid disk was observed. The control strains Klebsiella pneumoniae ATCC 700603 (ESBL positive) and *E. coli* ATCC 25922 (ESBL negative) were used for quality control.

### Statistical Analysis

All data was prepared and analyzed with the software WHONET 5.3, which is recommended by the World Health Organization. The *X*^2^ test was performed for comparing antibiotic and multidrug resistance proportions of *E. coli* strains using the SPSS version 13.0 software. Differences with a *X*^2^ *P* < 0.05 were considered statistically significant.

## Results

### Characteristics of *E. coli* Strains

A total of 100 *E. coli* strains were collected from January 2009 to December 2011 from neonates hospitalized at Beijing Children's Hospital. The clinical information of the neonates (including age and gender) from whom *E. coli* strains were isolated, as well as the source of the isolates in the present study, are reported in Table 1. A total of 78% of the strains were isolated from sputum samples, 10% from blood samples, 5% from CSF samples, and 7% from umbilical discharge.

**Table 1:** Clinical information of the neonates with isolated strains in the present study.

**Characteristics**	**No. of patients**
Gender	
Male	58 (58%)
Female	42 (42%)
Gestational age (weeks)	
28–37	5 (5%)
37–42	93 (93%)
>42	2 (2%)
Birthweight (g)	
<2,500	2 (2%)
2,500–4,000	89 (89%)
≥4,000	9 (9%)
Post-natal age (days)	
<7	24 (24%)
7–14	30 (30%)
14–21	21 (21%)
21–28	19 (19%)
>28	6 (6%)
Underlying diseases	
Meningitis	13 (13%)
Pneumonia	87 (87%)
Sepsis	27 (27%)
Patients' symptoms	
Fever	40 (40%)
Cough	44 (44%)
Jaundice	21 (21%)
Week response	43 (43%)
Convulsion	5 (5%)
Specimen types	
Sputum	78 (78%)
Venous blood	10 (10%)
Cerebroapinal fluid	5 (5%)
Umbilical discharge	7 (7%)
Outcome	
Cure	88 (88%)
Improve	12 (12%)
Unhealed	0

### Analysis of the Antimicrobial Susceptibility

The susceptibility of the *E. coli* strains to 10 antibiotics and the MICs of 100 *E. coli* isolates are presented in Table 1. Based on the CLSI 2016 criteria, the highest resistance rate of *E. coli* was to amoxicillin (85%), followed by cefuroxime (65%), and cefatriaxone (60%). Moreover, 6 and 5% of all isolates were resistant to amoxicillin/clavulanic acid and cefoperazone-sulbactam, respectively. The resistance rates to ceftazidime, gentamicin, ciprofloxacin, and sulfonamides were 31, 20, 33, and 47%, respectively. All isolates were susceptible to meropenem. More details about antimicrobial resistance rates are presented in Table 2.

**Table 2:** Susceptibility and MICs of 100 *E. coli* isolates to 10 antibiotics.

**Antibiotics**	**Susceptibility**			**MIC (μg/ml)**		
	**S (%)**	**I (%)**	**R (%)**	**50%**	**90%**	**Range**
Ceftazidime	63	6	31	1.5	>256	0.016–256
Cefuroxime	35	0	65	>256	>256	0.016–256
Cefatriaxone	37	3	60	>32	>32	0.002–32
Amoxicillin	9	6	85	>256	>256	1.0–>256
Meropenem	100	0	0	0.016	0.094	0.002–32
Gentamicin	73	7	20	0.75	24	0.016–256
Ciprofloxacin	67	0	33	0.19	>32	0.002–32
Cefoperazone-sulbactam	77	18	5	3	16	0.016–256
Amoxicillin-clavulanic acid	72	22	6	3	24	1.5–16
Sulfonamides	53	0	47	0.125	>32	0.002–32

### Multidrug-Resistant *E. coli*

The antibiotic resistance pattern of 100 *E. coli* isolates is shown in Table 3. Amoxicillin, cefuroxime, cefatriaxone, ceftazidime, amoxicillin-clavulanic acid, cefoperazone—sulbactam, and meropenem were classified as β-lactams. In contrast, gentamicin was classified as an aminoglycoside, while ciprofloxacin was regarded as a quinolone. We defined multidrug resistance in *E. coli* as resistance to at least three distinct antibiotic families and estimated this rate at ~26% (26/100) across all *E. coli* isolates.

**Table 3:** Antibiotic resistant pattern of 100 *E. coli* isolates.

**Class of antibiotic**	**Resistance pattern**	**No. of isolates**	**Proportion of all isolates**
0	–	5	5%
1	β-lactams	26	26%
2	β-lactams+aminoglycoside	5	5%
	β-lactams+quinolon	19	19%
	β-lactams+sulfonamides	19	19%
3	β-lactams+aminoglycoside+quinolon	4	4%
	β-lactams+aminoglycoside+sulfonamides	3	3%
	β-lactams+quinolon+sulfonamides	16	16%
4	β-lactams aminoglycoside+quinolon+sulfonamides	3	3%

Overall, the detection rate of ESBL-Producing *E. coli* was 55%. Specifically, this rate was significantly higher (*P* < 0.05) in sputum isolates (65%, 51/78) compared to aseptic humoral (27%, 4/15).

### Inspection Situation of ESBLs

The detection rate of ESBL-Producing *E. coli* was 55%. The rate in *E. coli* isolates from sputum (65%, 51/78) was higher than aseptic humoral (27%, 4/15). The difference was statistically significant (*P* < 0.05).

### Susceptibility of ESBL-Producing *E. coli* to Antimicrobial Agents

Importantly, the majority of the isolates were also resistant to non-β-lactam antimicrobial agents, even though the resistant rates were significantly lower than those observed in extended-spectrum β-lactamases. The differences between cefuroxime, cefatriaxone, and amoxicillin were statistically significant (*P* < 0.05), as shown in Table 4.

**Table 4:** Resistance rate of ESBL-Producing and non-ESBL-Producing *E. coli* strains.

	**ESBL**	**Non-ESBL**	***X^**2**^***	***P***
Ceftazidime	20 (36.4)	11 (24.4)	1.644	0.1998
Cefuroxime	51 (92.7)	14 (31.1)	41.303	0.00
Cefatriaxone	47 (85.5)	13 (28.9)	32.997	0.00
Amoxicillin	51 (92.7)	34 (75.6)	5.724	0.017
Meropenem	0	0	–	–
Gentamicin	12 (21.8)	13 (28.9)	0.66	0.417
Ciprofloxacin	19 (34.5)	14 (31.1)	0.09	0.764
Cefoperazone-sulbactam	3 (5.5)	3 (6.7)	0.064	0.8
Amoxicillin-clavulanic acid	5 (9.1)	6 (13.3)	0.455	0.5
Sulfonamides	23 (41.8)	23 (51.1)	0.86	0.354

### Clinical Treatment Condition of the Neonates With Isolated Strains in the Present Study

**Figure 2:**
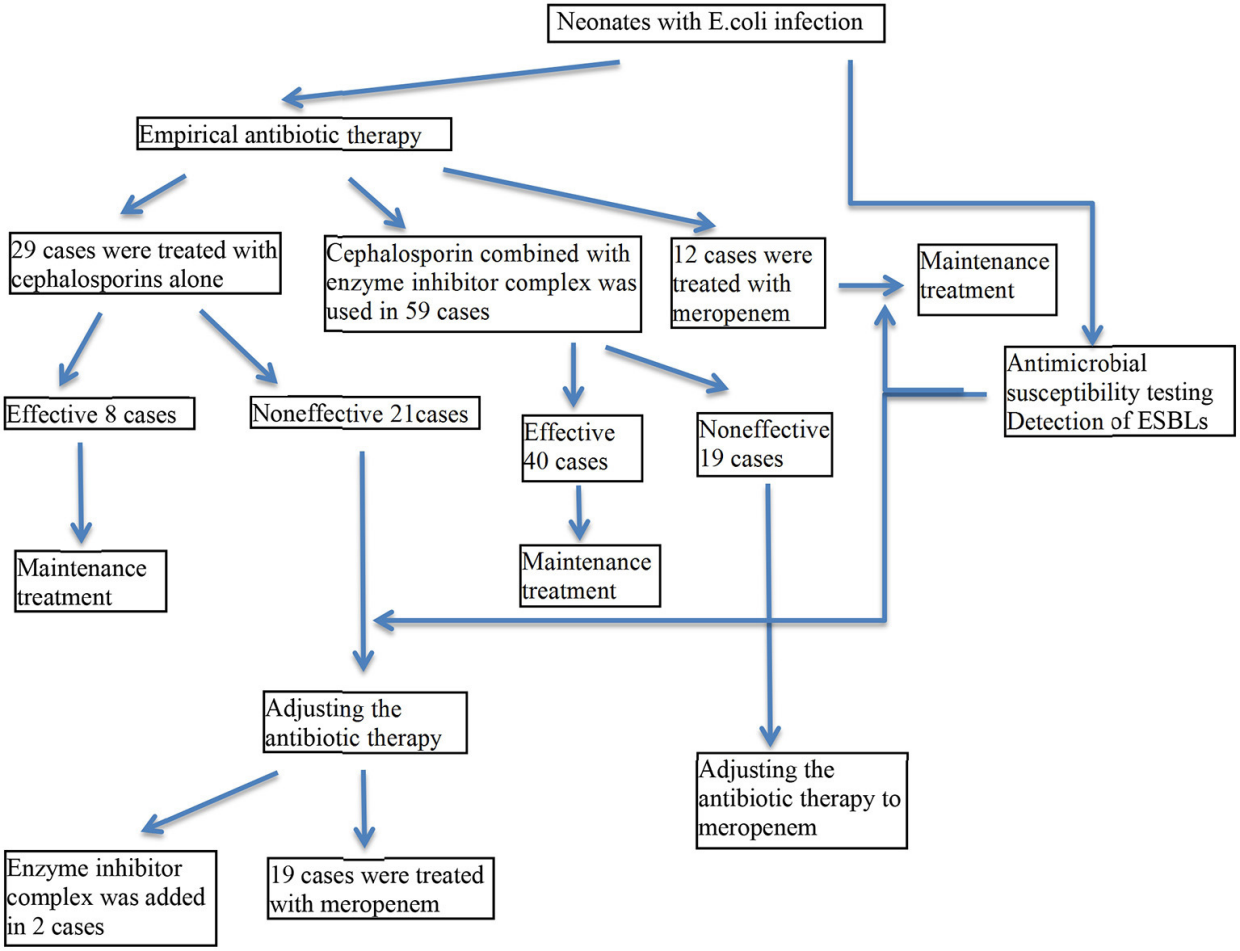
Treatment process of clinical neonates. The treatment procedures for all clinical neonates were shown.

The treatment process of clinical neonates is shown in Figure 2. The health conditions of all patients improved and some patients were completely cured by the end of the study.

## Discussion

*Escherichia coli* is the most frequent Gram-negative organism causing neonatal bacteremia and sepsis (8). Among febrile infants younger than 28 days-old, the prevalence of bacteremia and meningitis is high and most commonly caused by *E. coli* (9). The incidence of *E. coli* early-onset sepsis in very low birth weight infants was estimated at 1.04%, reaching a mortality rate of 35.3% (10). A recent meta-analysis based on a systematic review of published studies in the Chinese literature demonstrated that, among the newborn infants hospitalized in Chinese NICUs, ~50% of all *E. coli* bloodstream isolates (independently of being early or late onset) were multi-drug resistant due to extended-spectrum beta-lactamase (ESBL) production (11, 12).

Emerging antibiotic resistance is currently acknowledged as one of the most significant public health problems with high mortality rates associated with multidrug-resistant bacterial infections. The selective pressures imposed by antimicrobial use, overuse and misuse are driving the gradual increase in antibiotic resistance and leading to the emergence of multidrug-resistant bacterial strains. Previously treatable bacterial infections are now often untreatable or requiring the use of the last line of antibiotics (13). *E. coli* is the most common Gram-negative bacterial pathogen amongst resistant bacteria and causes a diverse range of diseases affecting all age groups. Multidrug-resistant, extensively drug-resistant and pan-drug-resistant strains of *E. coli* have now been reported worldwide, and this is becoming a critical global issue (14).

Cephalosporins belong to the β-lactam class of antibiotics and are presently the most commonly used antibiotics to treat gram-negative bacilli infection. *E. coli* strains can become resistant to beta lactam antibiotics by producing extended spectrum beta lactamase (ESBL), which is a plasmid-mediated β-lactamase that is capable of hydrolysing and inactivating β-lactams such as cephalosporins and monobactams (15). The identification of ESBL-producing *E. coli* (ESBL-*E. coli*) infections in infants in a neonatal intensive care unit is of particular concern because of the immature antibacterial immunity of neonates and the restricted therapeutic antibiotic options available (16).

The *E. coli* isolates often display resistance patterns that are typical of ESBL producers. In this study, the majority of the studied isolates showed resistance to amoxicillin (MIC range: 1.0–>256 mg/l), even though a very small proportion was resistant to a combination of amoxicillin with clavulanic acid (MIC range: 1.5–16). The antimicrobial resistance of experimental strains showed important differences between treatment with amoxicillin and amoxicillin-clavulanic acid. This indicates that while some *E. coli* strains can hydrolyze cephalosporins, this hydrolysis can be inhibited by clavulanic acid. A previous study had showed that these clinical *E. coli* isolates may produce group 2e β-lactamases (17). More recently, *E. coli* strains isolated from urine cultures of patients from Primary Care Barbastro Sector, between January 2011 and December 2013, showed a progressive increase to amoxicillin-clavulanate that reached 21.5% in 2013, a statistically significant increase (18) that was higher than the that presented in this study. Here, the *E. coli* strains isolated from the neonatal unit showed high resistance to amoxicillin, in accordance with the results found by Nitsch-Osuch et al. (19), which nevertheless described a relatively low degree of resistance to cephalosporins (1.8–5.3%) and aminoglycosides (0–2.6%) that were lower than our study. Bergin et al. (20) used multivariable logistic regression to evaluate the association between 30-day mortality and ampicillin-resistant *E. coli* bloodstream infections and were able to identify 123 (48%) ampicillin-resistant isolates. However, the authors found no significant association between ampicillin resistance and increased mortality, nor between antibiotic therapy and lower mortality.

Furthermore, Monsef et al. (21) reported a higher resistance of *E. coli* cultured from neonatal patients to cephalosporins and aminoglycosides. In our study, most *E. coli* isolates were resistant to cefuroxime (65 out of 100, MIC range: 0.016–256 μg/ml) and cefatriaxone (60 out of 100, MIC range: 0.002–32 μg/ml). Moreover, all isolates were susceptible to meropenem (MIC: <0.002 μg/ml), and the vast majority of the strains were susceptible to a combination of cefoperazone with clavulanic acid (MIC range: <0.016–32 μg/ml). We note that the majority of *E. coli* strains were also resistant to non-β-lactam antimicrobial agents, and some were resistant to sulfonamides (47 out of 100, MIC range: 0.002–32 μg/ml), ciprofloxacin (33 out of 100, MIC range: 0.002–32 μg/ml), and gentamicin (20 out of 100, MIC range: 0.016–256 μg/ml). As previously reported (22), tigecycline demonstrates excellent activity against a wide variety of Gram-positive and Gram-negative bacteria, including ESBL-producing organisms, and should thus be considered an encouraging antimicrobial agent. However, this antibiotic is not recommended in younger patients (<18 years of age) due to a lack of data regarding its safe usage, a problem that is potentially greater in the case of neonates (6).

Vernaz et al. (23) performed a retrospective observational time-series analysis to evaluate the incidence of non-duplicate clinical isolates of *E. coli* resistant to ciprofloxacin, trimethoprim/sulfamethoxazole and cefepime, from January 2000 through December 2007. The authors observed an increase in fluoroquinolone resistance among CA and HA isolates of *E. coli*, with slightly higher rates in the latter group, in accordance with data obtained from other European countries. They noted that the rate of ciprofloxacin resistance in *E. coli* is approaching the resistance rate of trimethoprim/sulfamethoxazole, and found that ciprofloxacin and cefepime resistance increased, Trimethoprim/sulfamethoxazole resistance remained stable, and total antibiotic use increased in both inpatient and outpatient settings. These results support efforts to reduce the prescription of fluoroquinolones for controlling resistant *E. coli*, including extended-spectrum β-lactamase producers.

In our study, we used meropenem as a treatment option to multidrug-resistant *E. coli* bacteremia. The recommended dosage of meropenem is calculated at 20 mg/kg q8h in neonates. Importantly, the use of meropenem in neonates warrants more concerns because of possible side effects such as anaphylaxis, liver and kidney impairment, and hemorrhagic symptoms. Even though all *E. coli* isolates from our study were sensitive to meropenem, it is not possible to fully evaluate the efficacy and safety of using carbapenems in pediatric patients, especially in neonates, and it is necessary to consider the existence of possible side effects. Meropenem and Imipenem are both members of carbapenems, a clinically important antibiotic family that is used in the treatment of Multidrug-Resistant (MDR) bacterial infections. However, susceptibility tests performed by the Kirby-Bauer disk diffusion method demonstrated that Imipenem sensitive *E. coli* BL21 cells overexpressing Ar-BVMO become resistant to this antibiotic. Agar disc diffusion assay further corroborates that, when Imipenem reacts with Ar-BVMO, it loses its antibiotic properties (24).

More than 25% of the isolates were resistant to at least three different classes of antibiotics in our study. A notable proportion showed cephalosporin resistant that probably reflects the epidemiology of ESBL-producing Enterobacteriaceae in China and the remainder of Asia.

Maternal treatment with antibiotics during pregnancy or at delivery should be considered as a possible influencing factor of *E. coli* neonatal resistance. In this study, three mothers had fungal vaginitis that was cured before pregnancy, so we expect this had no effect on the results observed here. In fact, we found a very close correlation between clinical manifestations and antibiotic resistance. All patients accepted our initial use of experimental anti-infection treatment, which was adjusted in the middle of the therapy according to the results obtained for the drug sensitivity test. All patients either improved or were cured, and were discharged from the hospital after successful therapeutic effects.

## Conclusion

Drug-resistant *E. coli* has become an important and complex problem in clinical treatment. This work reports a high rate of antimicrobial resistance, including ESBL positivity and multidrug resistance in a set of 100 *E. coli* isolates from neonates admitted into a large NICU. The data reported here are highly relevant for local antimicrobial prescription practice. In the future, this research can be expanded to increase sample size and type, and to enable evaluation of the correlation between clinical manifestations and antibiotic resistance. This newly available information about *E. coli* resistance in newborns will help informing clinical evaluation and decision-making.

## Data Availability Statement

The raw data supporting the conclusions of this article will be made available by the authors, without undue reservation.

## Author Contributions

YW designed the study. DW collected the data, analyzed the data, and wrote the first draft of the manuscript, which was significantly edited by YW. YD, KY, and WG were participants in the workshop and the round-table and either gave presentations. All authors read and approved the final manuscript.

## Conflict of Interest

The authors declare that the research was conducted in the absence of any commercial or financial relationships that could be construed as a potential conflict of interest.

## Abbreviations

*E. coli*: *Escherichia coli*
ESBL: Extended-spectrum beta-lactamase
NICU: Neonatal intensive care unit
ExPEC: Extraintestinal pathogenic *E. coli*
CSF: Cerebrospinal fluid.
